# The evolution of mini-chromosomes in the fungal genus *Colletotrichum*

**DOI:** 10.1128/mbio.00629-23

**Published:** 2023-06-07

**Authors:** Haoming Wang, Rong Huang, Jingyi Ren, Lihua Tang, Suiping Huang, Xiaolin Chen, Jun Fan, Bintao Li, Qinhu Wang, Tom Hsiang, Huiquan Liu, Qili Li

**Affiliations:** 1 State Key Laboratory of Crop Stress Biology for Arid Areas, College of Plant Protection, Northwest A&F University, Yangling, Shaanxi, China; 2 Institute of Plant Protection, Guangxi Academy of Agricultural Sciences, Key Laboratory of Green Prevention and Control on Fruits and Vegetables in South China Ministry of Agriculture and Rural Affairs and Guangxi Key Laboratory of Biology for Crop Diseases and Insect Pests, Nanning, Guangxi, China; 3 College of Natural Resources and Environment, Northwest A&F University, Yangling, Shaanxi, China; 4 School of Environmental Sciences, University of Guelph, Guelph, Ontario, Canada; University of Georgia, Athens, Georgia, USA; Vanderbilt University, Nashville, Tennessee, USA

**Keywords:** mini-chromosomes, pathogenicity, evolution, *Colletotrichum*, genome, transcriptome

## Abstract

Anthracnose diseases caused by *Colletotrichum* species are among the most common fungal diseases. These symptoms typically manifest as dark, sunken lesions on leaves, stems, and fruit. In China, mango anthracnose seriously affects fruit yield and quality. Genome sequencing of several species shows the presence of mini-chromosomes. These are thought to contribute to virulence, but their formation and activity remain to be fully elucidated. Here, we assembled 17 *Colletotrichum* genomes (16 isolated from mango plus one from persimmon) through PacBio long-read sequencing. Half of the assembled scaffolds had telomeric repeats at both ends indicating full-length chromosomes. Based on comparative genomics analysis at interspecies and intraspecies levels, we identified extensive chromosomal rearrangements events. We analyzed mini-chromosomes of *Colletotrichum* spp. and found large variation among close relatives. In *C. fructicola*, homology between core chromosomes and mini-chromosomes suggested that some mini-chromosomes were generated by recombination of core chromosomes. In *C. musae* GZ23-3, we found 26 horizontally transferred genes arranged in clusters on mini-chromosomes. In *C. asianum* FJ11-1, several potential pathogenesis-related genes on mini-chromosomes were upregulated, especially in strains with highly pathogenic phenotypes. Mutants of these upregulated genes showed obvious defects in virulence. Our findings provide insights into the evolution and potential relationships to virulence associated with mini-chromosomes.

**IMPORTANCE:**

*Colletotrichum* is a cosmopolitan fungal genus that seriously affects fruit yield and quality of many plant species. Mini-chromosomes have been found to be related to virulence in *Colletotrichum*. Further examination of mini-chromosomes can help us elucidate some pathogenic mechanisms of *Colletotrichum*. In this study, we generated novel assemblies of several *Colletotrichum* strains. Comparative genomic analyses within and between *Colletotrichum* species were conducted. We then identified mini-chromosomes in our sequenced strains systematically. The characteristics and generation of mini-chromosomes were investigated. Transcriptome analysis and gene knockout revealed pathogenesis-related genes located on mini-chromosomes of *C. asianum* FJ11-1. This study represents the most comprehensive investigation of chromosome evolution and potential pathogenicity of mini-chromosomes in the *Colletotrichum* genus.

## INTRODUCTION

Mango (*Mangifera indica*) is one of the most commercially significant fruit crops worldwide. Mango anthracnose caused by several *Colletotrichum* species has been considered the most important disease on mango in southern China (1). Fungi within the genus *Colletotrichum* can be subdivided into species complexes, which are comprised of closely related species (2). The *Colletotrichum gloeosporioides* species complex (CGSC) contains pathogens that can infect a wide range of hosts and cause significant damage (3). *C. asianum* in CGSC is the dominant species causing mango anthracnose in China (4).

Fungal pathogens have highly dynamic genomes that show high variability in size and composition even within closely related species, primarily due to genomic rearrangements and differences in repetitive DNA content (5). The phenomenon of transposon propagation occurred in the evolution of *Colletotrichum* genomes, and transposon content varies significantly among species, ranging from 4.3% in *C. scovillei* to 44.9% in *C. orbiculare* (6). Compared with shared genomic regions, transposon content of strain-specific regions is higher (7). Transposon propagation results in increases in genome size but the number of protein-coding genes remains stable (8, 9).

In recent years, reference genomes of several *Colletotrichum* strains have been assembled from long-reads generated by third-generation sequencing, and the scaffold lengths have been significantly improved over second-generation short-read assemblies. The genome of *C. higginsianum* IMI349063 was sequenced using PacBio technology combined with optical mapping data (10, 11), to yield 28 contigs among 12 chromosomes, and a contig N50 of 5.20 Mb, and the genome assembly was considered greatly improved compared with previous versions (12). Recently, *C. fructicola* strain 1104-7 isolated from apple (13), *C. fructicola* Nara gc5, several *C. siamense*, and *C. aenigma* strains isolated from strawberry were sequenced using third-generation technology and assembled. These assemblies were close to complete chromosomal level (14). Despite the abundance of genomes sequenced in *Colletotrichum*, there are still many species that have not been sequenced.

Many pathogenic fungi have evolved mini-chromosomes, also known as accessory chromosomes. These mini-chromosomes are unrelated to their vegetative growth (15), less than 2 Mb, and are significantly different from core chromosomes in genomic characteristics such as gene density, transposon content, sequence variation, and GC content. The available evidence shows that mini-chromosomes exist in pathogenic fungi such as *Fusarium oxysporum* (16, 17), *Magnaporthe oryzae* (18 - 20), *Botrytis cinerea* (21), and *Alternaria alternata* (22).

In members of the *Colletotrichum* genus, mini-chromosomes were first discovered by using pulse electrophoresis technique (23). Sequences of mini-chromosomes #11 and #12 of *C. higginsianum* were revealed through resequencing using PacBio single-molecule real-time (SMRT) sequencing (11). The mini-chromosomes are rich in effector proteins and some genes of unknown function but the overall gene ratio is significantly lower than core chromosomes. The GC content of mini-chromosomes is low, and 40% of mini-chromosome #11 of *C. higginsianum* is composed of transposons (11). Analysis of effector candidate genes showed that they were not randomly distributed on the *C. higginsianum* genome but distributed near the telomeric regions and arranged in clusters (12). A sequenced isolate of *C. higginsianum* which lacks mini-chromosome #11 showed weak pathogenicity on *Arabidopsis thaliana* (24). However, these studies have not revealed the how mini-chromosomes are generated nor located specific virulence factors on mini-chromosomes.

In this study, we report PacBio genome sequencing data of 16 representative *Colletotrichum* strains isolated from mango, and 1 from persimmon (*Diospyros* sp.), and analysis of RNA-seq data of 6 dominant strains from mango. We performed comparative genomics analysis across the *Colletotrichum* genus at interspecies and intraspecies levels using our sequenced genomes plus ones available on the NCBI database. The potential origin and expression of pathogenesis-related genes on mini-chromosomes were fully investigated. We confirmed that mini-chromosomes could encode virulence genes by transcriptomic evidence and phenotypes of mutants.

## MATERIALS AND METHODS

### PacBio genome sequencing and assembly

Sixteen strains were isolated from mango leaves with anthracnose symptoms collected from the six mango-growing provinces in China (4), and additional strain PLG3-2 was isolated from persimmon fruit in Guangxi, China (Table 1). The genomic DNA of collected strains was extracted from pure cultures using the CTAB method (25). Libraries were prepared using the the SMRTbell Template Prep Kit, and sequenced using the PacBio Sequel II instrument all following PacBio protocols. The libraries were sequenced on three sMRT cells using the P6-C4 polymerase-chemistry at 50, 65, and 75 pM DNA concentrations. The subreads generated from PacBio platform were corrected and trimmed using FALCON v.1.3.0 (26) and further assembled with both FALCON and MECAT2 (27). Quickmerge v.0.3 (28) was used to integrate the assembly results of the above assemblers to generate more continuous genome drafts. FinisherSC v.2.1 (29) was used to scaffold the contigs generated by Quickmerge. The genome sequences were polished with original subreads using arrow (https://github.com/PacificBiosciences/GenomicConsensus). The long reads were mapped to assemblies to identify palindromic structures that might have been generated by incorrect assembly. IGV (30) was used to manually confirm mis-assembled contigs, and they were removed from subsequent analyses. Merqury v.1.3 (31) was then used to evaluate the completeness and accuracy of assembled genomes. The telomeric repeat sequence “TTAGGG” was located at the end of scaffolds using customized Python script (available at https://github.com/AlexWanghaoming/mango).

**TABLE 1:** Information on sequenced isolates of *Colletotrichum* species collected in China for this study

Strain	Species	Host	Collection location	Species complex
YN55-1	*C. asianum*	*Mangifera indica*	Yunnan Province	*gloeosporioides*
FJ11-1	*C. asianum*	*Mangifera indica*	Fujian Province	*gloeosporioides*
HN47-2	*C. fructicola*	*Mangifera indica*	Hainan Province	*gloeosporioides*
QZ-3	*C. fructicola*	*Mangifera indica*	Guangxi Province	*gloeosporioides*
GD10-1	*C. siamense*	*Mangifera indica*	Guangdong Province	*gloeosporioides*
YN56-1-1	*C. siamense*	*Mangifera indica*	Yunnan Province	*gloeosporioides*
GZ15-1	*C. gloeosporioides*	*Mangifera indica*	Guizhou Province	*gloeosporioides*
GZ14-2	*C. fructicola*	*Mangifera indica*	Guizhou Province	*gloeosporioides*
HN32-1	*C. tropicale*	*Mangifera indica*	Hainan Province	*gloeosporioides*
HN42-2	*C. gigasporum*	*Mangifera indica*	Hainan Province	*gigasporum*
YN31-4	*C. cliviae*	*Mangifera indica*	Yunnan Province	*orchidearum*
YN32-6	*C. endophytica*	*Mangifera indica*	Yunnan Province	*gloeosporioides*
YN33-1	*C. liaoningense*	*Mangifera indica*	Yunnan Province	*magnum*
YN51-1	*C. scovillei*	*Mangifera indica*	Yunnan Province	*acutatum*
HN23-5	*C. cordylinicola*	*Mangifera indica*	Hainan Province	*gloeosporioides*
GZ23-3	*C. musae*	*Mangifera indica*	Guizhou Province	*gloeosporioides*
PLG3-2	*C. horii*	*Diospyros* sp.	Guangxi Province	*gloeosporioides*

### Identification and characterization of repetitive elements

RepeatModeler v.1.0.11 (http://www.repeatmasker.org) was first used to *ab initio* predict the repetitive sequences in the genome. The predicted repetitive sequences were integrated with the RepBase (32) database, and RepeatMasker v.4.0.9 (33) was used to identify the repetitive sequences in each genome by searching for them in the repetitive database. LTR_FINDER v.1.07 (34) and LTRharvest v.1.5.9 (35) were used to predict the full-length LTR retrotransposons separately. LTR_retriever v.2.8.7 (36) was then used to merge the results and obtain advanced full-length LTR retrotransposons.

### Illumina RNA library preparation and sequencing

The most frequently collected species of *Colletotrichum* (GZ15-1, GD10-1, FJ11-1, YN55-1, YN56-1-1, and HN47-2) were grown on potato dextrose agar (PDA) and on mango leaves. For isolates GZ15-1, FJ11-1, YN55-1, and HN47-2, hyphae on PDA and spots on mango leaves were collected after 5 days of growth. For YN56-1-1 and GD10-1, conidia and spots after 3 days were collected. Illumina strand-specific RNA-seq libraries were sequenced on the Illumina HiSeq 2500 system to yield 2 × 150 bp paired-end reads. The target for the number of raw paired-end sequencing reads from each strain was more than 40 million, and with total number of bases more than 10 Gb.

### RNA-seq analysis

RNA-seq reads were quality-trimmed using fastp v.0.20.1 (37). Clean reads were then mapped to their respective genome using Hisat v.2.1.0 (38). FeatureCount v.1.5.1 (39) was then used to count reads mapped to coding regions as a measure of expression level. TPM normalization was conducted using in-house scripts (available at https://github.com/AlexWanghaoming/mango). Differentially expressed genes at the infection stage were identified using edgeR (40) with “FDR<0.05”, “fold change>2” as threshold values. Gene model prediction requires assembled transcripts and information on intron position. Therefore, stringTie v.2.1.3b (41) was used to assemble RNA-seq reads and generate transcripts. STAR v.2.7.2b (42) was used to calculate junction sites of introns.

### Gene prediction

Gene model prediction was conducted with multiple steps. We first employed BRAKER2 pipeline (43) , which integrates evidence from GeneMark-ES v.4.48_3.60 (44) *ab initio* prediction, transcriptome and protein homology, and sends these results to AUGUSTUS v.3.3.1 (45) for gene models prediction. Second, we used the GeneMark-ES suite alone for gene model prediction. GeneMark_ET (46) which integrates junction site information was used to predict gene models. Next, we extracted open reading frames in assembled transcripts using TransDecoder v.5.5.0 (47). Finally, according to the assigned weights, all the above gene prediction results and transcript evidence were integrated into EVidenceModeler v.1.1.1 (48) to obtain the final gene prediction models. BUSCO v.3.0.2 (Benchmarking Universal Single-Copy Orthologs) (49) software and fungal single-copy homologous gene set (fungi_odb10) were used to evaluate the completeness of the predicted gene sets of all strains.

### Gene annotations

Gene functional annotation was performed, and gene ontology (GO) terms of all proteins were assigned using Interproscan v.5.45-80.0 (50). GO enrichment analysis was performed using ClusterProfiler (51). The online software dbCAN2 (http://bcb.unl.edu/dbCAN2) was used to predict carbohydrate-active enzymes (CAZymes) proteins identified by two of three embedded toolkits (HMMER, Diamond, Hotpep) and these were defined as CAZymes. The online toolkit antiSMASH (52) was used to predict secondary metabolite biosynthetic gene clusters. SingalP v.5.0 (53) and DeepLoc v.1.0 (54) were employed for signal peptide prediction and subcellular localization. Proteins with signal peptides located extracellularly were defined as secreted proteins and these composed the secretome separately by isolate. EffectorP v.2.0 (55) was used to predict effectors. The gene sequences in the secretomes were used in BLASTp v.2.7.1 to search for homologous secreted proteins in the NCBI non-redundant protein database (*E*-value < 1 × 10^−5^). Putative secreted proteins with homology to proteins in the genus *Colletotrichum* were defined as candidate secreted effector proteins (CSEPs).

### Comparative genomic analysis

Orthogroups (orthologs and paralogs) from the 26 genomes of *Colletotrichum* genus (17 assembled in this study plus 9 downloaded from NCBI) were obtained using OrthoFinder v.2.5.1 (56). Multiple sequence alignments of single copy genes were extracted, and TrimAl v.1.4.1 (57) was used to remove regions with low conservation in multiple sequence alignments. PhyML v.3.3 (58) was used to construct phylogenetic trees using the LG amino acid substitution model and the maximum likelihood algorithm. Phylogenetic trees were visualized using R package ‘ggtree’ with midroot method which takes midpoint of the two taxa with the longest path on the tree as the root. Nucmer program in the MUMmer v.3.1 software (59) was used to align the genomic sequence of seven dominant strains of *Colletotrichum* and generate syntenic blocks. Following the method stated in previous study (60), adjacent blocks with predicted distances smaller than 20,000 bp were connected using custom Python scripts (available at https://github.com/AlexWanghaoming/mango) to obtain larger syntenic blocks, and to display whole genomic synteny profiles and identify chromosomal rearrangement events. JCVI (61) was used to infer the syntenic gene pairs among strains, and among seven strains, genomic regions that appeared in less than three strains were identified as lineage-specific (LS), and those found in at least six strains were defined as conserved genomic regions.

### Genome resequencing analysis

In total, 27 *C*. *asianum* strains were collected in China and subjected to Illumina short-read DNA sequencing at the Beijing Genomics Institute (Shenzhen, China). The clean reads were aligned to the YN55-1 reference genome using BWA v.0.7.17-r1188 (62) software, and bcftools v.1.10.2 (63) was used to identify SNPs. “Two-speed” refers to a bipartite genome architecture of filamentous pathogen, in which the fast subgenome is responsible for adaption and infection (64). The unique SNPs were used to infer two-speed genomic regions. R package mixtools (65) was used to fit a Gaussian mixture model . The EM (Expectation Maximization) algorithm was used to solve the model’s parameters. The hidden Markov states of each interval were determined by using the R package depmixS4 (66).

### Chromosomal karyotype analysis

The germ tube burst method (GTBM) (67) was used for karyotype analysis. The spores of *Colletotrichum* strains were first collected and adjusted to 7.5 ×10^4^ cells/mL with potato dextrose broth (PDB). About 150 µL of spore suspension was added dropwise to a poly-L-lysine-coated slide and incubated for 12–13 h in a dark, humid chamber at 28°C to allow germination. PDB was then removed with a pipette, and 150 µL of PDB containing 100 µg/mL thiabendazole was placed on the slide and incubated for another hour. The slides were then washed with distilled water, and some ultrapure water was kept on the slide to keep the sample moist. The slide was placed horizontally in fixative solution (methanol:glacial acetic acid=9:1) and left for more than 20 min at room temperature. The fixed slides were flame-dried and stored at room temperature until use. Before observing the sample, 2× SSC liquid containing 100 µg/mL RNase A was added to the slide and left for 1 h at 37°C, then stained with 1 µg/mL 4′,6-diamidino-2-phenylindol dihydrochloride (DAPI), and sealed with a glass coverslip. Samples were observed with an Olympus BX53/DP80 fluorescence microscope at 1,000× magnification.

### Deletion and complementation of gene CASFJ_18279

Homologous reorganization was used to delete the CASFJ_18279 gene in *C. asianum* (FJ11-1). The 0.53 kb upstream and 1.19 kb downstream flanking fragments of CASFJ_18279 were amplified with primer pairs 18279F1S+18279F1A and 18279F2S+18279F2A (Table S6). The resultant PCR products were fused with the hygromycin resistance cassette remodeled from pMD18-T (68). The recombinant plasmid was introduced into *Agrobacterium tumefaciens* following a high-efficiency ATMT transformation system (68). Hygromycin (Beijing Solarbio Technology Co., Ltd, China) was added to the final concentration of 100 µg/mL for transformant selection. Transformants resistant to hygromycin were screened by PCR with primer pairs Δ18279F1S+Δ18279F1A, Δ18279F2S+Δ18279F2A, and Δ18279S+Δ18279A to confirm the deletion of CASFJ_18279.

To construct the complementation vector, the 2986 bp CASFJ_18279 fragment, containing a 1446 bp upstream, a full-length CASFJ_18279 gene coding region without termination codon, and an 828 bp downstream, and was amplified from genomic DNA of wild-type strain FJ11-1 using primers 18279comS/18279comA (Table S6) and cloned into pNeo3300III (69). The complementation vector was transformed using ATMT by co-culture with the ΔCASFJ_18279 strain (70). Since the mutant ΔCASFJ_18279 did not grow on PDA amended with antibiotic G418 (Solarbio Life Sciences (Beijing) Co., Ltd. Beijing, China), the neomycin resistance cassette was chosen as the selectable marker for the complementation transformation. Putative complementation mutants with neomycin resistance were selected for PCR using primers Δ18279comS/Δ18279comA (Table S6). The complemented strains were then analyzed for pathogenicity.

### Pathogenicity and plant infection assays

Pathogenicity and virulence tests were performed on freshly harvested mango fruits (cv. Tainong) without visible disease. Mango fruits were first dipped in 75% alcohol for 10 s, surface sterilized in 2% sodium hypochlorite for 1 min, and rinsed three times with sterile water. The fruits were allowed to dry (or dried with autoclaved paper to welling) and placed into plastic boxes, each containing five fruits. The mutant ΔCASFJ_18279, complemented strains, and wild-type strain were used to test pathogenicity, and 15 fruits were inoculated per strain. Hyphal plugs (6 mm diam) from the growing margin of PDA cultures were inoculated on the lightly wounded fruits, and the control group was treated with sterile PDA. After inoculation, a thin layer of sterile water was misted on the surface of the leaves, and the plants were covered with plastic bags to maintain high humidity and incubated at 28°C. The development of symptoms was observed daily, and the lesion sizes on each leaf in two perpendicular directions were measured 7 days post-inoculation to assess the virulence.

## RESULTS

### Seventeen high-quality *Colletotrichum* genomes generated from PacBio sequencing

In order to construct high-quality *Colletotrichum* genome sequences, we collected strains and conducted PacBio sequencing of seven dominant strains (*C. asianum* YN55-1, *C. asianum* FJ11-1 *C. fructicola* HN47-2, *C. fructicola* QZ-3, *C. siamense* YN56-1-1, *C. siamense* GD10-1, and *C. gloeosporioides* GZ15-1) that cause mango anthracnose and 10 non-dominant strains isolated from mango and persimmon (Table 1 and Table S1). For each strain, we generated 1.29 Mb long-reads with a length of 5,928 to 11,694 bp on average. The average sequencing depth was over 280× (Table S1). After genome assembly of the 17 strains, the numbers of scaffolds ranged from 9 to 21 and average scaffold N50 was higher than 4.39 Mb (Table S1). These strains had an average of 49% scaffolds with telomere repeats “TTAGGG” at both ends. According to previous studies, the number of chromosomes of *Colletotrichum* is usually 10–13, which is close to the number of scaffolds assembled in this study for each genome (average 14), and hence the assemblies of these genomes are considered chromosomal level. We evaluated the base accuracy and completeness of these genome sequences using a *k*-mer based method (Merqury), and the results showed that the base accuracy of the *Colletotrichum* genome assemblies was higher than 99.99%. These results indicated that these *Colletotrichum* genome sequences assembled by PacBio sequencing in this study were highly representative and continuous, which are of great value for comparative genomics studies.

### Genome and gene features of *Colletotrichum*

**Fig 1:**
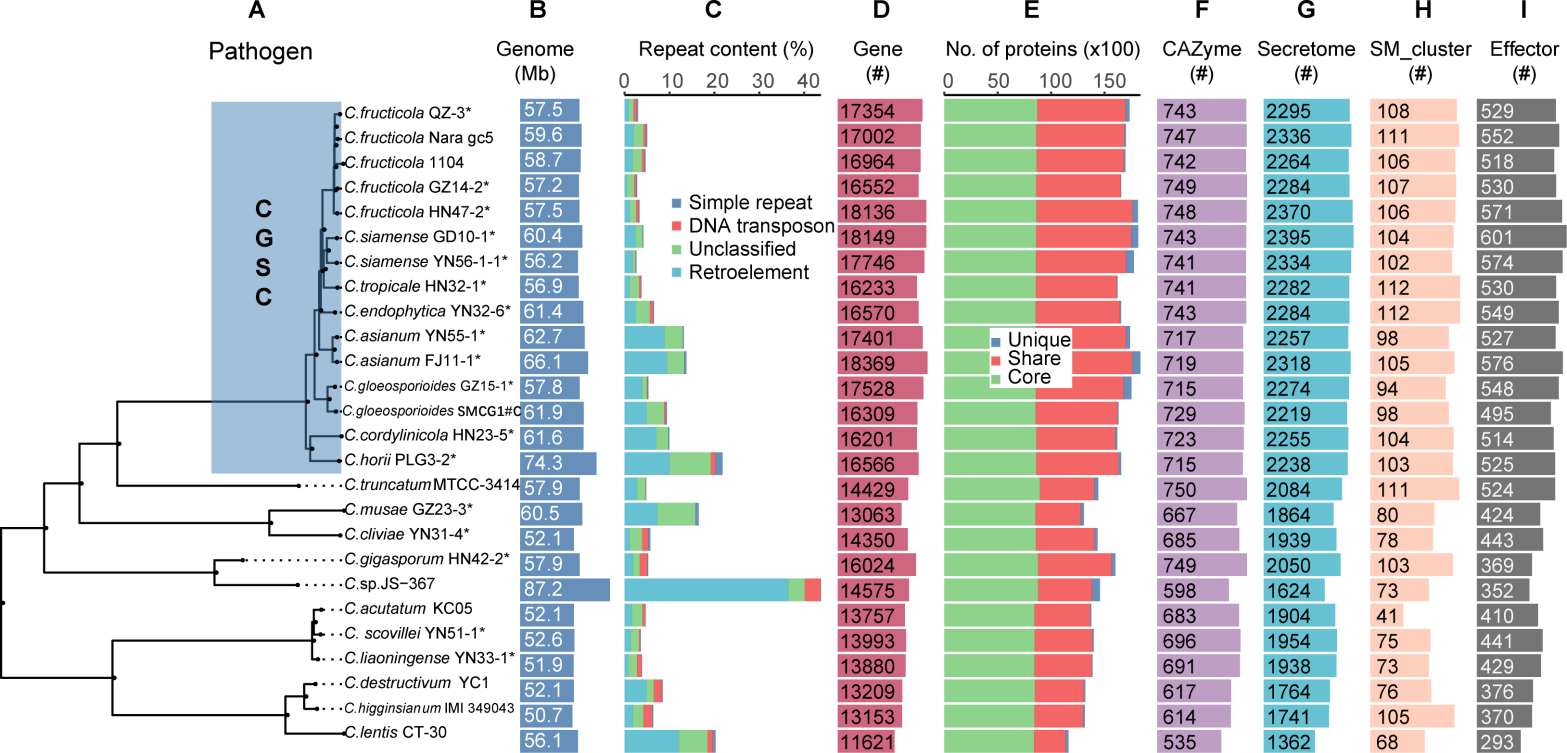
Phylogenomic relationship and gene annotation of *Colletotrichum* strains. (**A**) Maximum likelihood phylogenomic tree was constructed using 6,461 single-copy genes/orthogroups. CGSC is highlighted using blue rectangle. Asterisks represent strains sequenced in this study. (**B**) Genome size. (**C**) Percentage of four marked categories of repeats. (**D**) Number of protein-coding genes. (**E**) Number of protein-coding genes belonging to orthologue families conserved in all (Core) or a subset (Shared) of *Colletotrichum* species or species/strain-specific (Unique). Number of predicted carbohydrate-active enzymes (CAZY) (**F**), secreted proteins (Secretome). (**G**) Gene clusters related to secondary metabolite (SM) biosynthesis (**H**) and predicted effector proteins (**I**). CGSC, *Colletotrichum gloeosporioides* species complex.

To better understand the characteristics of genomes of *Colletotrichum*, we obtained sequences of an additional nine genomes from the NCBI genome database. In total, 26 genomes of *Colletotrichum* strains (19 species) were used for comparison. The average size of the nuclear genome sequence of these 26 genomes was 59.04 Mb. Among them, *C*. sp. JS-367 had the largest genome at 87.2 Mb and *C. higginsianum* had the smallest genome at 50.7 Mb (Fig. 1B). These results indicate that genomes of different *Colletotrichum* strains may vary greatly.

**Fig 2:**
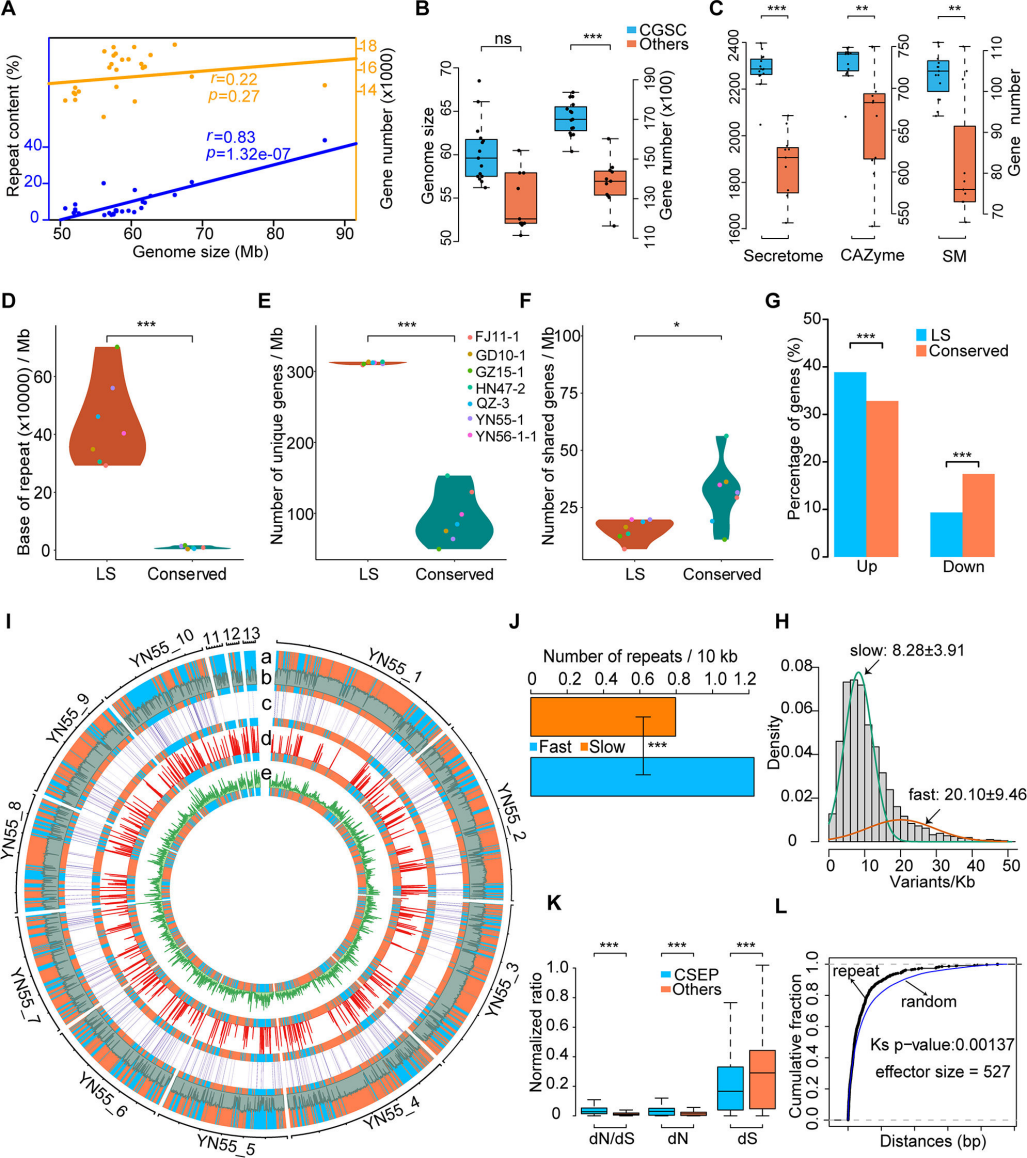
Landscape of genomic features of CGSC strains. (**A**) Correlation between genome size and repeat content or gene number. *r* denotes Pearson correlation coefficient. (**B**) Comparison of genome size and gene number. (**C**) Numbers in Secretomes, CAZymes, and SM clusters. (**D–F**) show the length of repeat sequences, number of strain-specific genes, and number of shared genes in the conserved and LS=lineage specific genomic regions. (**G**) Bar-plot display the enrichment of genes upregulated during infection in the LS genomic regions of strain GD10-1. (**H**) Histogram of variant density distributions for YN55-1 based on a bin of 10 kb. The curves illustrate the two Gaussian distributions estimated based on a Gaussian mixture model using the Expectation-Maximization algorithm. (**I**) Circos plot of genomic features of *Colletotrichum asianum* YN55-1. Concentric circles show different features of YN55-1 that were drawn in 10 kb non-overlapping windows; a: fast and slow-evolving genomic regions, blue and orange rectangles represent fast- and slow-evolving regions respectively; b: GC content; c: heatmap of secreted proteins density; d: distribution of repeat density; and e: distribution of SNPs from 27 other *C. asianum* strains. (**J**) Repeat content of fast and slow-evolving genomic regions. (**K**) The non-synonymous (dN) or synonymous (dS) substitution rate and dN/dS ratio of CSEP genes and other genes, respectively. (**L**) Cumulative distribution curve of absolute distance between effector genes and repeat elements. *, *P *< 0.05; **, *P* < 0.01; ***, *P *< 0.001; and ns, not significant. Statistical significance was assessed by one-sided Wilcoxon (**A, B, D, E, F, K**), χ^2^ test (**G, J**), and Kolmogorov-Smirnov test (**L**). CGSC, *Colletotrichum gloeosporioides* species complex; CSEP, candidate secreted effector protein; SM, secondary metabolite.

Repetitive sequences of the 26 *Colletotrichum* strains were then identified using a uniform pipeline, and the average repetitive sequence content was found to be 8.91%. *C.* sp. JS-367 and *C. siamense* GD10-1 had the most (43.77%) and the fewest (2.65%) repetitive sequences, respectively (Fig. 1C). Pearson correlation test results showed that the genome sizes of *Colletotrichum* strains were positively correlated with the content of repetitive sequences (*r* = 0.83, *P* value <0.001, Fig. 2A).

We then performed protein-coding gene prediction for 26 *Colletotrichum* genomes, and the completeness based on predicted genes reached more than 99% as evaluated by BUSCO (Table S2). Gene prediction results showed that gene counts of *Colletotrichum* ranged from 11,621 (*C. lentis* CT-30) to 18,369 (*C. asianum* FJ11-1) (Fig. 1D). Strains belonging to *C. gloeosporioides* species complex had more predicted genes than those belonging to other species complexes although their genome sizes did not show obvious difference (Fig. 2B). The results of the Pearson correlation test showed that the gene count was not significantly related to the genome size (*r* = 0.22, *p* value = 0.27, Fig. 2A).

Gene family analysis of the protein-coding genes revealed that *Colletotrichum* genomes encoded 22,147 orthologous gene families, containing 402,583 genes, which accounted for 98.2% of all predicted genes (409,885). Among them, 8,114 gene families were shared by all 26 strains and were therefore defined as the core gene families. Some core gene families showed expansion. For example, *C. gigasporum* HN42-2 had 8,785 predicted core genes (Fig. 1E), which was higher than the average number 8,599 in all species. In addition, *Colletotrichum* strains evolved their own strain-specific genes, ranging from 31 (*C. fructicola* GZ14-2) to 731 (*C.* sp. JS-367), accounting for 0.2% to 5.0% of the total number of predicted genes.

We performed phylogenetic analysis of these 26 strains based on all 399,364 genes belonging to 21,943 orthogroups (Fig. 1A). Previous studies have shown that *C. higginsianum*, *C. destructivum,* and *C. lentis* belong to the *destructivum* species complex (71). *C. scovillei* YN51-1 belongs to the same clade as *C. acutatum* (72). *C. liaoningense* YN33-1 belongs to the *magnum* species complex which is closer to *C. acutatum* species complex*. C. truncatum* MTCC-3414 belongs to the *truncatum* species complex. The precise identity of *C.* sp. JS-367, an endophyte obtained from mulberry (GenBank QCWU00000000.1), is still unknown. Although *C.* sp. JS-367 placed closest to *C. gigasporum* HN42-2 (Fig. 1A), an ultrametric tree indicated that the time of divergence was 20.08 million years ago (Fig. S2A). Moreover, LTR retrotransposons account for 36.53% of genomic sequences in *C.* sp. JS-367, which was the highest among all classes of repetitive sequences. All intact LTR retrotransposons of *C.* sp. JS-367 arose after its divergence from *C. gigasporum* HN42-2 (Fig. S2B).

The CAZymes, secreted proteins, effectors, and secondary metabolite synthesis gene clusters in the genomes of each strain were identified and analyzed (Fig. 1F–I). We found that 15 strains belonging to CGSC had an average of 734 CAZymes, 105 secondary metabolite biosynthesis gene clusters, and 2,294 secreted proteins. The numbers of these potentially pathogenic genes were significantly higher than the average of that in non-CGSC strains (662 carbohydrate active enzymes, 80 secondary metabolite biosynthesis gene clusters, and 1,839 secreted proteins) (Fig. 2C).

### Characteristics of lineage-specific (LS) and fast-evolving genomic regions

Some filamentous pathogens evolved LS genomic regions and a fast-evolving subgenome. Genes in these regions contribute to pathogenicity (60, 73, 74). In order to identify LS and fast-evolving genomic regions in *Colletotrichum* genomes, we performed synteny analysis of seven dominant strains from mango anthracnose (*C. asianum* FJ11-1, *C. asianum* YN55-1, *C. siamense* GD10-1, *C. siamense* YN56-1-1, *C. fructicola* HN47-2, *C. fructicola* QZ-3, and *C. gloeosporioides* GZ15-1). The synteny profile (Fig. S1) showed that most genomic regions of the seven strains were conserved. However, due to the loss of genome fragments or the existence of repetitive sequences, some syntenic breakpoints were still distributed on each chromosome. For example, compared to the reference strain GD10-1, the GZ15-1 strain lacked bases at positions 1 to 536,564 bp on chromosome #5 (Fig. S1). The long-reads coverage generated from PacBio sequencing provided strong evidence of these syntenic breakpoints. We further defined the regions that appeared in the genomes of less than three strains as LS genomic regions, and the regions that appeared in the genomes of at least six strains were defined as conserved genomic regions. The results indicated that the LS genomic regions were rich in repetitive sequences (Fig. 2D). In addition, LS regions had more strain-specific genes and fewer shared genes (genes present in at least two strains) compared to the conserved genomic region (Fig. 2E–F), and LS genomic regions were also rich in genes that were upregulated during the infection stage (Fig. 2G). These results implied that the LS regions were closely related to the genomic diversity and pathogenicity, and that LS regions were of great significance for the generation of novel genes and species adaptations.

We then used *C. asianum* as a model to investigate intraspecies variation and analyze the two-speed genomic features (64) in *Colletotrichum*. We sequenced genomes of 28 strains of *C. asianum* that were collected in China. Using the genome of the YN55-1 strain as a reference, 172,338 SNP loci were identified. We fit the Gaussian mixture model and obtain a two-speed genome structure, where there were fast-evolving portions and slow-evolving portions. The average mutation frequency of fast-evolving genome region was 20.1 ± 9.5 bases/kb, and the average variation frequency of slow-evolving regions was 8.3 ± 3.9 bases/kb (Fig. 2H).

The analysis of the two-speed genomes showed that the fast-evolving portion represented only 34% (Fig. 2I), and that up to 90% of the LS genomic regions overlapped with the fast-evolving genomic regions. Furthermore, fast-evolving genomic regions harbored more secreted proteins and repetitive sequences (Fig. 2I and J). We identified the CSEPs in the *Colletotrichum* genomes. Positive selection analysis revealed that the dN/dS and non-synonymous mutation (dN) of CSEPs were significantly higher than that of other genes (Fig. 2K), indicating that they were under strong natural selection pressure. By comparing the distance between effector genes and repetitive sequences in the YN55-1 strain, we found that the effector genes were spatially closer to the repetitive sequences than random genomic regions (Fig. 2L).

### Extensive chromosomal rearrangements have occurred in *Colletotrichum* genomes

**Fig 3:**
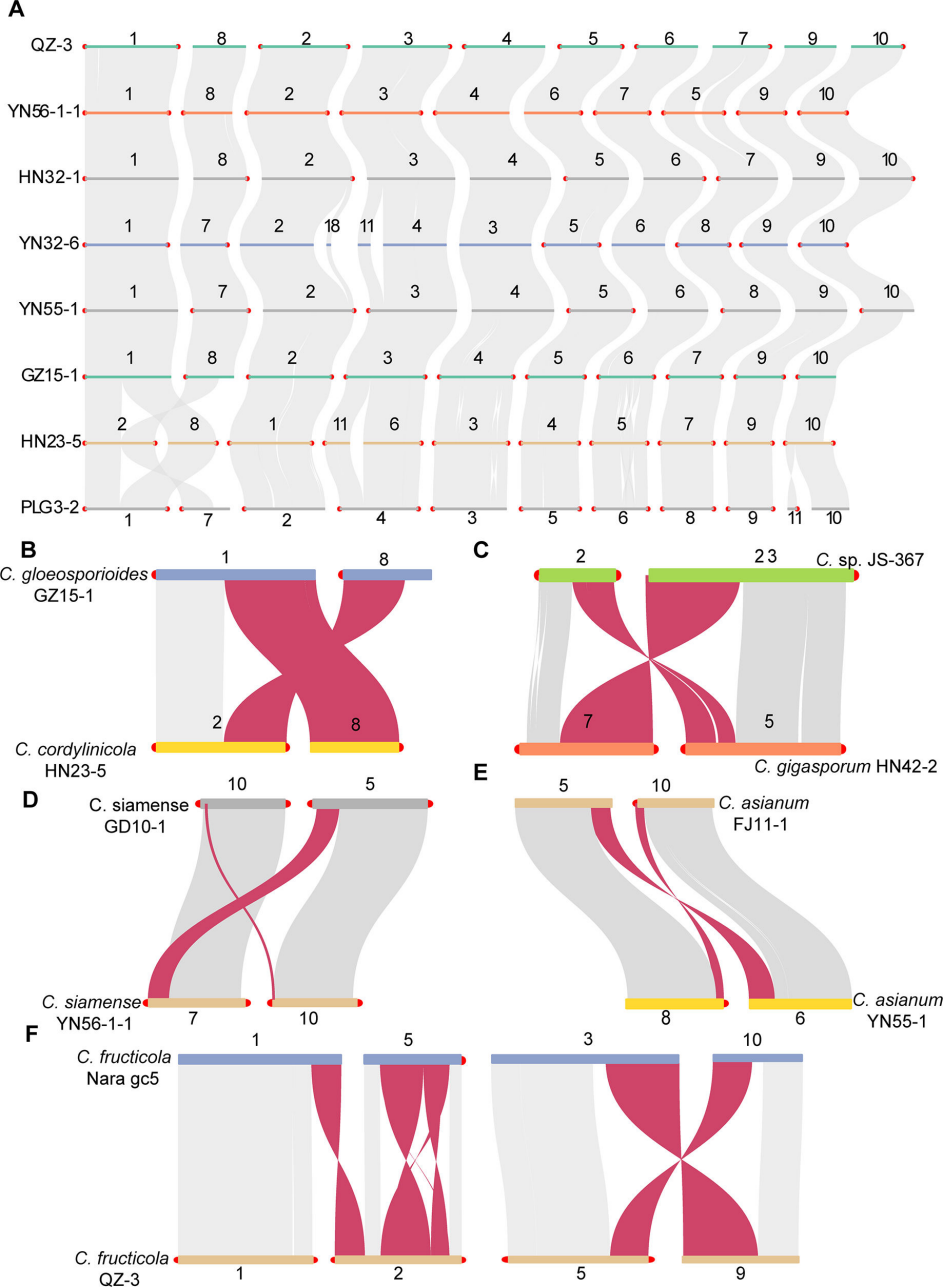
Macro-synteny plot and chromosomal rearrangement events of representative strains in CGSC. Interspecific (**A–C**) and intraspecific (**D–F**) chromosomal rearrangement events. CGSC, *Colletotrichum gloeosporioides* species complex.

Syntenic analysis of the genomes of the CGSC showed that 10 core chromosomes were found in different species (Fig. 3A). We observed extensive chromosomal rearrangement events on core chromosomes. For example, in strain HN23-5 (*C. cordylinicola*), chromosomes #2 and #8 have been rearranged and contain portions homologous to chromosomes #1 and #8 of *C. gloeosporioides* GZ15-1 strain (Fig. 3B). Outside of the CGSC, chromosomes #2 and #23 of *C.* sp. JS-367 showed homology with chromosomes #5 and #7 of HN42-2 (*C. gigasporum*) (Fig. 3C). Furthermore, a chromosomal fusion event occurred in the genome of YN31-4 (*C. cliviae*), which resulted in only nine core chromosomes (Fig. S3).

We also performed intraspecies genomic synteny analysis, and results showed that chromosomal rearrangement events were observed in *C. siamense*, *C. asianum*, and *C. fructicola.* For example, in *C. siamense*, chromosomes #10 and #5 of GD10-1 showed homology to chromosomes #7 and #10 of YN56-1-1 (Fig. 3D). In *C. asianum*, chromosomes #5 and #10 of FJ11-1 showed homology with chromosomes #6 and #8 of YN55-1 (Fig. 3F). In *C. fructicola,* chromosomal rearrangements occurred more frequently (Fig. 3E) than for other species, especially for strain Nara gc5 which showed many rearrangements compared to other strains of this species.

### The mini-chromosomes of *Colletotrichum* are widespread and highly specific

**Fig 4:**
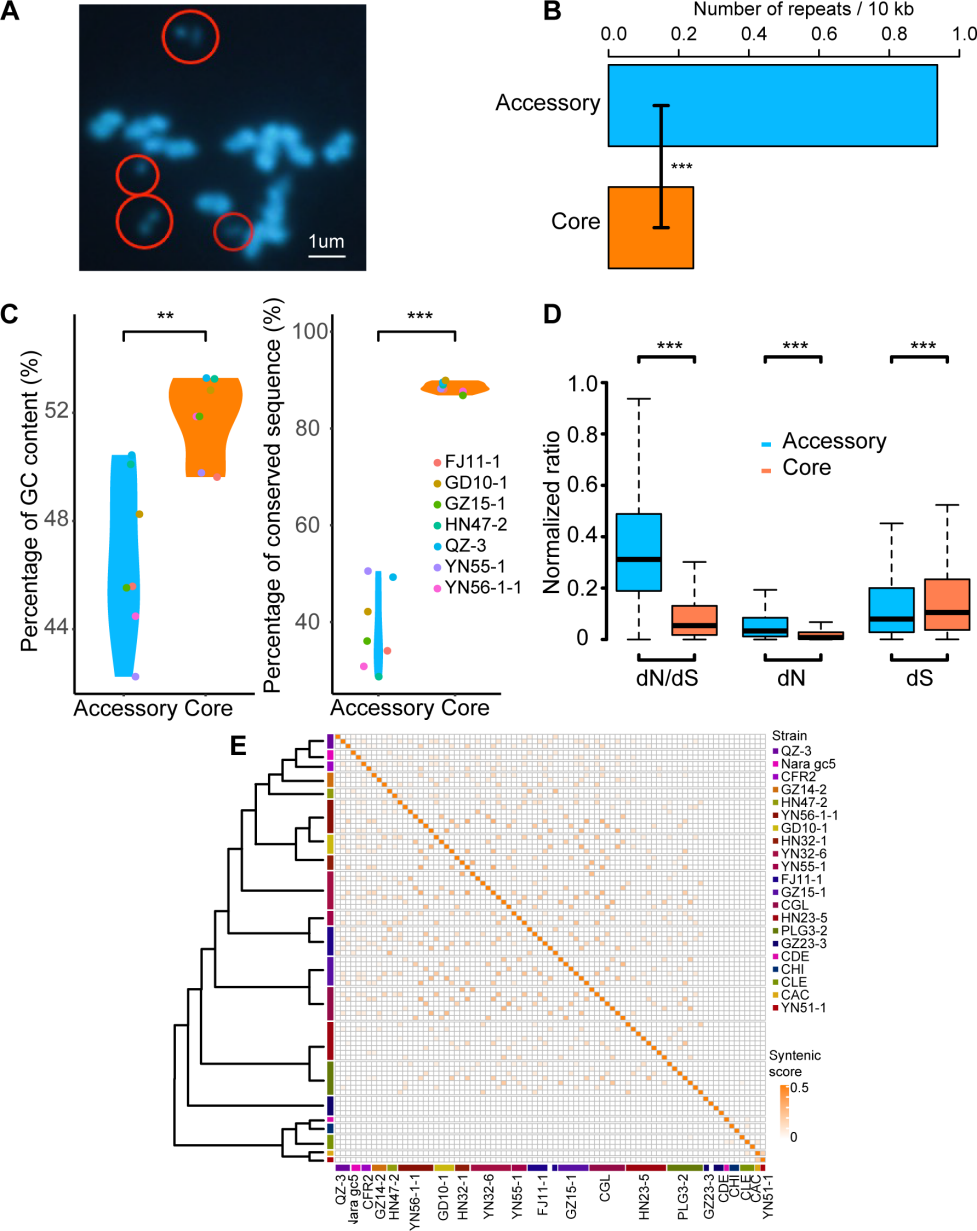
Characteristics of mini-chromosomes. (**A**) Karyotype map of chromosomes of *Colletotrichum gloeosporioides* GZ15-1 obtained by the germ tube burst method. The scale bar is 1 µm. Red circles enclose mini-chromosomes. (**B**) Repeat content on core and mini-chromosomes per 10 kb. (**C**) Percentage of GC content, conserved sequence in mini-and core chromosomes, respectively. (**D**) The non-synonymous (dN) or synonymous (dS) substitution rate and dN/dS ratio of genes on mini-and core chromosomes. (**E**) Topology structure of phylogenomic tree of strains which harbored mini chromosomes and heatmap of syntenic score matrix. Synteny score for each strain pair was estimated using formula, $Syntenic\;score\left(a,b\right)=\left(N_{a}+N_{b}\right)/\left(N_{a}\times N_{b}\right)$ , where *a* and *b* represent two strains, and $N_{a}$ and $N_{b}$ represent the number of total syntenic genes on their syntenic blocks, respectively. CGL, CAC, CDE, CHI, and CLE denote *C. gloeosporioides* SMCG1#C, *C.* sp. JS-367, *C. acutatum* KC05, *C. destructivum* YC1, *C. higginsianum* IMI349043, and *C. lentis* CT-30, respectively. **P < 0.01; ***P < 0.001. Statistical significance was assessed by χ^2^ test (**B**) and one-sided Wilcoxon tests (**C, D**).

In addition to the conserved core chromosomes, CGSC also had some mini-chromosomes (also known as accessory chromosomes). The numbers of these mini-chromosomes ranged from three to eight per genome (Table S4). Many mini-chromosomes had telomeric repeats at both ends, indicating that they were complete chromosomes. Non-CGSC strains also had mini-chromosomes, except for YN31-4, JS-367, HN42-2, YN33-1, or MTCC-3414. The strains with the most mini-chromosomes were YN32-6 (*C. endophytica*) and HN23-5 (*C. cordylinicola*), both of which had eight mini-chromosomes. The strains with the fewest mini-chromosomes were *C. acutatum* KC05, *C. destructivum* YC1 and YN51-1 (*C. scovillei*), with only one mini-chromosome each. Moreover, *C. lentis* had the longest mini-chromosome with a length of 1.52 Mb, and HN23-5 (*C. cordylinicola*) had the shortest mini-chromosome with a length of 0.27 Mb (Table S4). In order to rule out the possibility that these sequences of mini-chromosomes were caused by assembly errors, we used *C. gloeosporioides* GZ15-1 as the model to perform chromosomal karyotype analysis. The results showed that the number of mini-chromosomes assembled matched the number of mini-chromosomes observed under a fluorescence microscope (Fig. 4A).

We then compared the GC content and sequence similarity of mini and core chromosomes. We found that the mini-chromosomes had more repetitive sequences (Fig. 4B), with a lower proportion of GC content and fewer conserved sequences (Fig. 4C). The functions of most genes found on mini-chromosomes are still unknown. dN/dS analysis showed that these genes were under strong selection pressure (Fig. 4D), indicating that genes on mini-chromosomes evolved more rapidly than genes on core chromosomes. In order to illustrate whether there were conserved accessory regions among different species, gene similarity analysis among 26 strains was carried out (Fig. 4E). The results showed that a few genes on mini-chromosomes of CGSC strains were similar to each other. However, we did not find homologous genes outside the CGSC, showing that the mini-chromosomes might have evolved independently in different species complexes and were related to the adaptive evolution of individual species complexes.

### Generation of mini-chromosomes from recombination of core chromosomes

**Fig 5:**
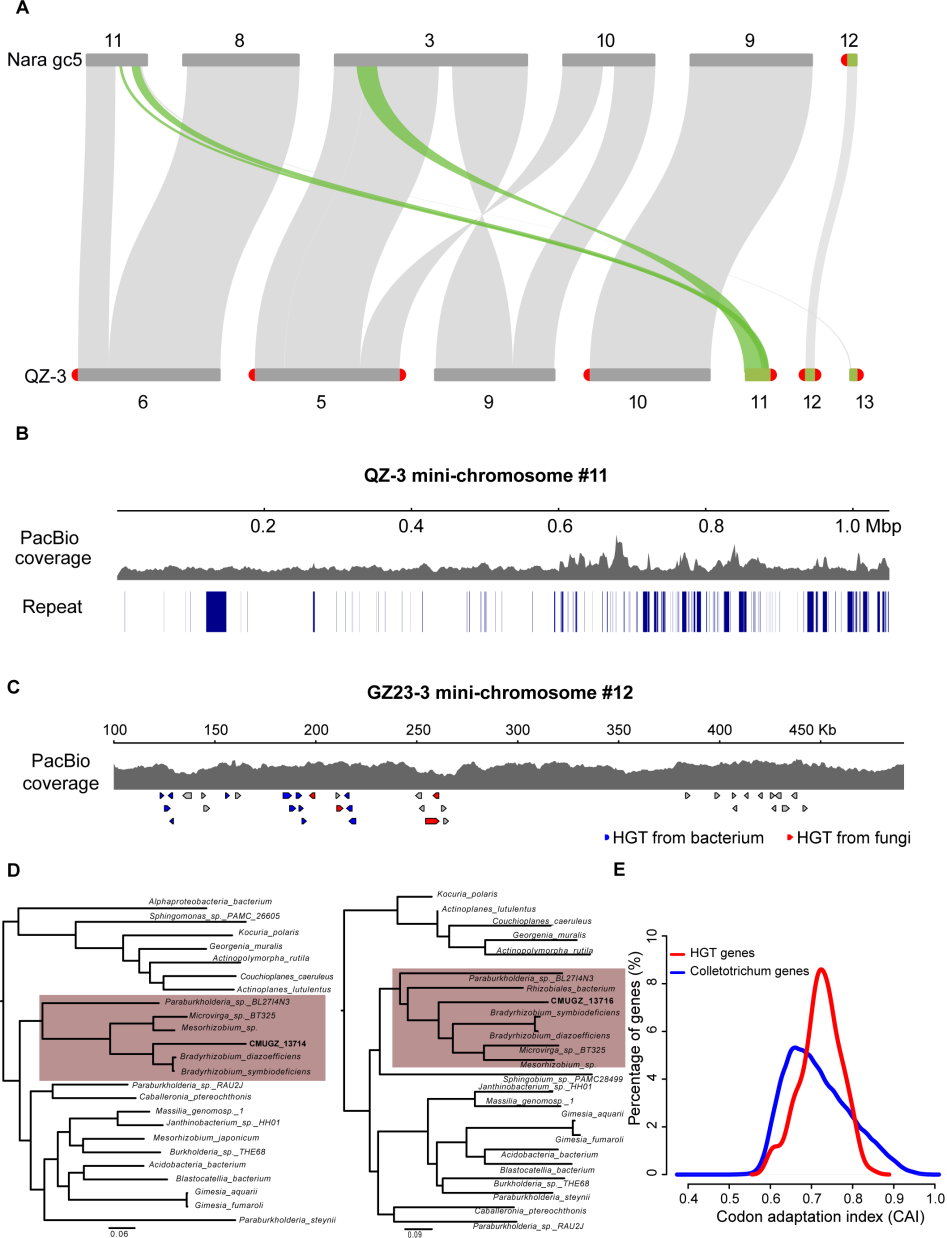
Generation and evolution of Mini-chromosomes of *Colletotrichum*. (**A**) Mini-chromosome #11 of QZ-3 derived from interspecific chromosomal rearrangement. Green ribbons represent homologous sequences. (**B**) PacBio coverage and repeat sequences of *C. fructicola* QZ-3 mini-chromosome #11. (**C**) HGT genes on chromosome #12 of GZ23-3 strain. Blue and red arrows denote genes transferred from bacterium and fungi, respectively. (**D**) Phylogenetic tree of two representative HGT genes. (**E**) The CAI distribution of HGT genes compared to *Colletotrichum* genes. The CAI derived from the RSCU estimations is computed using the EMBOSS tool “cai.” HGT, horizontal gene transfer.

In *M. oryzae*, it has been found that sequences with a length of 761 kb on a mini-chromosome were highly similar to segment a on core chromosome a, suggesting a possible origin of the accessory chromosome from a core chromosome (20). We conducted intraspecies genomic synteny analysis and found a sequence of approximately 590 kb on the mini-chromosomes #11 of *C. fructicola* QZ-3 to be highly similar to a region of core chromosome #3 of *C. fructicola* Nara gc5 (Fig. 5A). This region consisted of 184 genes. To determine their origin, we aligned this sequence against the core chromosomes of every strain and found that the region from the mini-chromosome had the highest similarity (99.4%) to positions 3,548,799–4,135,098 of chromosome #5 of QZ-3 itself. This implied that the mini-chromosome fragment was derived from a core chromosome in the same genome. Interestingly, in QZ-3, a short fragment near the telomeric repeat of mini-chromosome #11 was similar to a fragment of core chromosome #11 of Nara gc5. This fragment happened to be missing in core chromosome #6 of QZ-3. Mini-chromosome #11 of QZ-3 was assembled in a complete sequence without any breakpoints (Fig. 5B). These results provided evidence that mini-chromosome #11 of QZ-3 was generated by the recombination of its own core chromosomes #5 and #6.

### Some genes on mini-chromosomes come from horizontal gene transfer

To further study the origin of genes on mini-chromosomes, we searched for homologous proteins in the NCBI non-redundant protein database. We found that 162 genes were most similar to genes in non-*Colletotrichum* species. We thus suspected they were obtained by horizontal gene transfer (HGT). Among them, *C. musae* GZ23-3 had the largest number of potentially HGT genes on its mini-chromosomes, with 37 genes, 26 of which matched those from bacterial genomes, and the rest showed homology to other fungal genomes. Further analysis of the 26 genes in GZ23-3 showing homology to bacterial genes indicated that they showed high similarity to bacterial genes related to ATP binding (Table S5), and were arranged in clusters on mini-chromosomes #12 and #13 (Fig. 5C). Phylogenetic analysis showed that two examples (CMUGZ_14714 and CMUGZ_13716) were shared by the bacterial order Rhizobiales and were most similar to genes of *Bradyrhizobium* (Fig. 5D). Potentially HGT genes showed different codon usage preferences compared to genes of *Colletotrichum* itself (Fig. 5E).

### Pathogenesis-related genes on mini-chromosomes are upregulated during infection

To illustrate functions of genes on mini-chromosomes, we conducted RNA-seq analysis of six dominant strains causing mango anthracnose (*C. asianum* FJ11-1, *C. asianum* YN55-1, *C. siamense* GD10-1, *C. siamense* YN56-1-1, *C. fructicola* HN47-2, and *C. gloeosporioides* GZ15-1) (Table S3) and analyzed their gene expression profile. Compared to the hyphal stage, an average of 2,375 genes were upregulated at 5 days after infection in each strain. Among these upregulated genes, an average of 15 genes were located on mini-chromosomes (Table 2).

**TABLE 2:** Differentially expressed gene counts on mini-chromosomes at the infection stage compared to vegetative hyphal stage

Gene counts	YN56-1-1	GD10-1^*b*^	HN47-2	YN55-1	FJ11-1^*c*^	GZ15-1
Total genes	18,149	17,746	18,136	17,401	18,369	17,528
Total genes upregulated >2-fold at 3 dpi	2,152	668	–	–	–	–
Total genes upregulated >2-fold at 5 dpi	2,530	2,383	1,700	2,011	3,048	2,580
Potentially pathogenic genes^*a*^ upregulated >2-fold at 5 dpi	674	629	543	452	682	659
Genes located on mini-chromosomes	470	236	483	191	882	372
Genes located on mini-chromosomes upregulated >2-fold at 3 dpi	1	0	–	–	–	–
Genes located on mini-chromosomes upregulated >2-fold at 5 dpi	4	7	5	3	51	17
Potentially pathogenic genes located on mini-chromosomes upregulated >2-fold at 5 dpi	0	1	4	1	10	4

^*a*^ Potential pathogenic genes comprise of secreted proteins, CAZymes and secondary metabolite biosynthetic gene clusters.

^*b*^ GD10-1 showed higher pathogenic compared to YN56-1-1 according to a previous report (4).

^*c*^ FJ11-1 showed higher pathogenic compared to YN55-1 according to a previous report (4).

**Fig 6:**
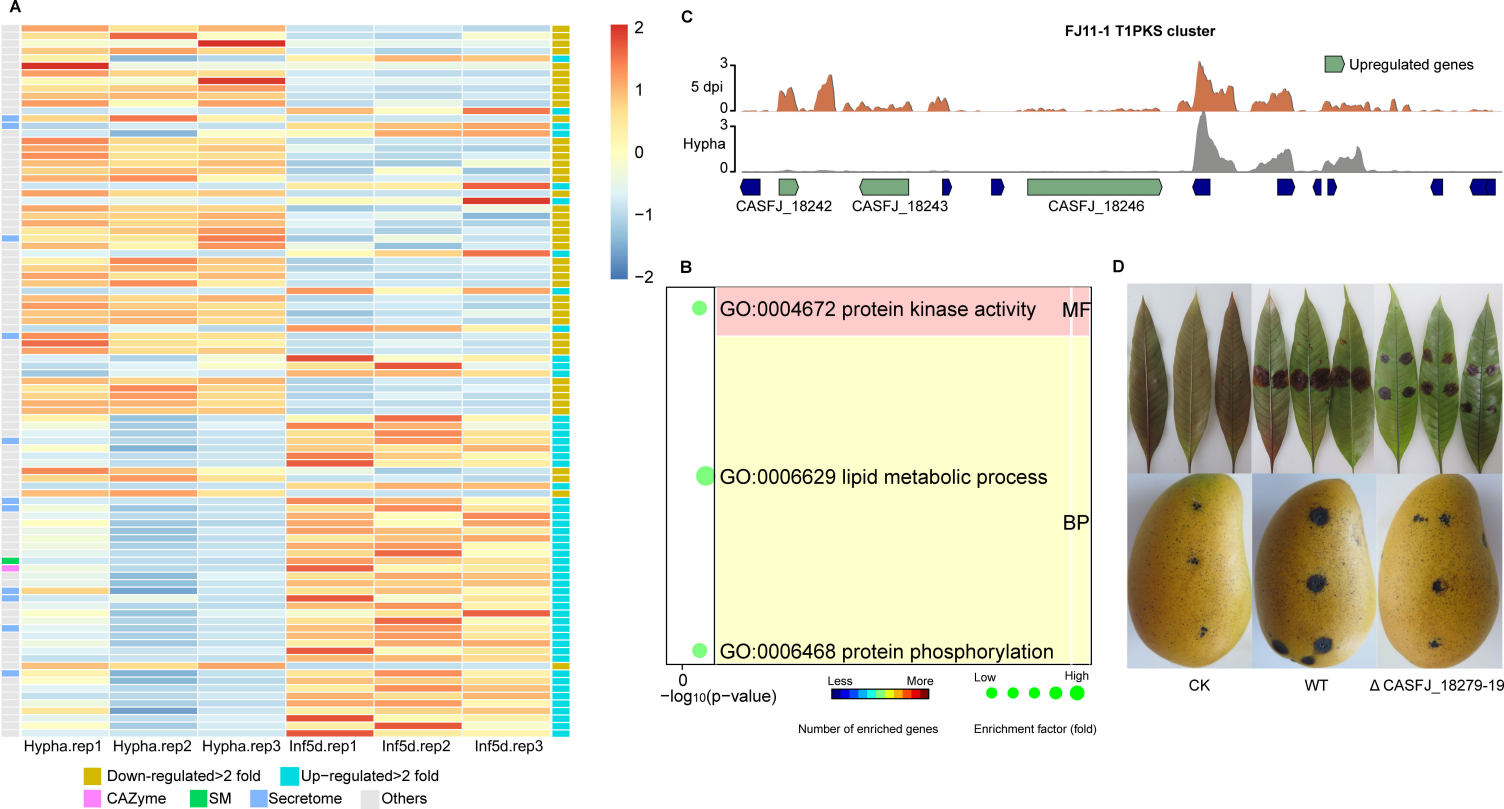
Gene expression profile *in planta* compared to *in vitro* hyphae of potentially pathogenesis-related genes on mini-chromosomes. (**A**) Heatmap of differentially expressed genes on mini-chromosomes of strain FJ11-1. Potential pathogenesis-related genes are marked. (**B**) GO enrichment analysis of genes upregulated >2-fold. (**C**) Expression of T1PKS cluster of FJ11-1 mini-chromosome. 5 dpi, 5 days post-inoculation. (**D**) ΔCASFJ_18279-19 mutant inoculated on mango leaves and fruit. CK (check) showed no obvious symptoms after 3 days at 28°C. GO, gene ontology.

Previous study showed that strains FJ11-1 and GD10-1 were more aggressive than their closest relative such as YN55-1 and YN56-1-1 in this study (4). Compared to isolates YN55-1 and YN56-1-1, FJ11-1 and GD10-1 had more potential pathogenic genes located on mini-chromosomes (Table 2). Among upregulated genes located on mini-chromosomes of FJ11-1, eight genes encoded secreted proteins and one was an SM gene (Fig. 6A). GO enrichment analysis showed that upregulated genes were related to protein kinase activity and phosphorylation (Fig. 6B) which are crucial for infection by pathogenic fungi (75 - 77). We further investigated the expression of T1PKS (type I polyketide synthase) genes on FJ11-1 mini-chromosome #16, which were highly expressed 5 days after inoculation. Among them, CASFJ_18242, CASFJ_18243, and CASFJ_18246 were upregulated during the infection stage (Fig. 6C). The T1PKS cluster is specific to strain FJ11-1. To validate the function of genes on mini-chromosomes, we knocked out gene CASFJ_18279 which was upregulated during the infection stage and was identified only on mini-chromosome of FJ11-1. All three mutants (ΔCASFJ_18279-19, ΔCASFJ_18279-22, and ΔCASFJ_18279-33) showed reduced aggressiveness compared to wild-type on both mango leaves and fruit (Fig. 6D; Fig. S4). The complementation strain of mutants exhibited normal pathogenicity identical to the wild-type (Fig. S4). These results indicated that genes on these mini-chromosomes may contribute to pathogenicity of *C. asianum* FJ11-1.

## DISCUSSION

Although many *Colletotrichum* species have been sequenced and their genome sequences have been published, existing genomic resources are far from comprehensive relative to the diversity of the genus *Colletotrichum.* In this study, we sequenced and assembled 17 *Colletotrichum* genomes using long-read technology, and the genomes of some of these species (*C. endophytica* YN32-6, *C. liaoningense* YN33-1, and *C. cordylinicola* HN23-5) are the first reports. Our assembled genomes were representative with distribution in the five species complexes, and continuous with half of the scaffolds showing telomeric repeats at both ends. These genome sequences will enrich the existing *Colletotrichum* genome resources. A wide range of comparative genomic analyses of 26 strains (19 species and 7 species complexes) were performed, and similar to a previous report (14), strains belonging to CGSC had more genes than non-CGSC strains. Moreover, CGSC strains were abundant in potentially pathogenic genes such as secreted proteins, CAZymes, and secondary metabolite biosynthetic gene clusters.

The presence of transposons is usually detrimental to the organism, but in pathogenic fungi, transposons are associated with pathogenic genes, and the rapid evolution of these regions drives the mutation of pathogenic genes resulting in enhanced adaptation of pathogenic fungi (5). Interspecies comparisons identified LS genomic regions that were enriched in transposon sequences and strain-specific genes. Extensive LTR burst events occurred in the genome of *C.* sp. JS-367 after it diverged from *C. gigasporum* HN42-2, resulting in high variation between the two. Furthermore, the two-speed evolutionary nature of *Colletotrichum* genomes was revealed by intraspecies comparisons. The rapidly evolving genomic regions were also enriched in transposon sequences and secreted proteins, which may be associated with the pathogenicity of the strain. The high concordance (90%) between the LS and fast-evolving genomic regions implied that transposons were important for adaptive evolution and genomic diversity in the *Colletotrichum* genomes. Additionally, extensive chromosomal rearrangements among strains are likely to be induced by active transposon insertions (78, 79). However, these transposons and rapidly evolving genomic regions did not alter the number of core chromosomes in *Colletotrichum*, and genomic syntenic analysis showed that *Colletotrichum* genomes commonly possess 10 core chromosomes and 0–8 mini-chromosomes.

In this study, we performed systematic characterization of mini-chromosomes of *Colletotrichum* genomes and found that mini-chromosomes were common. Gene function annotation methods based on homologous sequence alignment and structural domain searches revealed that the function of most genes on the mini-chromosomes remains unknown, perhaps because genes on the mini-chromosome underwent strong positive selection and thus random mutations have occurred, resulting in lack of matches to existing databases. We also found that some genes from the fast-evolving genomic region on the core chromosomes could have integrated into mini-chromosomes. Moreover, core chromosomes of *C. fructicola* QZ-3 underwent rearrangement to form a novel mini-chromosome. These results demonstrate that mini-chromosomes may be generated by core chromosome recombination. In addition, HGT genes from or to bacteria were found on the mini-chromosome of strain GZ23-3, which were arranged in clusters and had functions such as ATP-binding. These examples did not appear to be common across strains, but such analyses are hampered by the difficulty of accurately tracing the origin of these rapidly evolving genes and chromosomal fragments by homologous alignment methods. Furthermore, these findings point the way for continued studies on the origin of mini-chromosomes.

The existence of mini-chromosomes was discovered 20 years ago with the advent of pulsed electrophoresis technology (80). But only in recent years have several studies demonstrated the association of mini-chromosomes with pathogenic fungal pathogenesis (24, 81). However, these studies only performed experiments on genetic mapping localization or chromosome deletion or acquisition at a macroscopic level and did not analyze the origin and function of genes on mini-chromosomes. We observed in this study that within the same species, strains with highly pathogenic phenotypes may possess multiple potentially pathogenic genes located on mini-chromosomes. A PKS cluster was only found on a mini-chromosome of strain FJ11-1 with a highly pathogenic phenotype, whereas it was absent in the strain YN55-1 with a lower pathogenic phenotype. These genes were copied from the fast-evolving genomic region of a core chromosome and displayed a high expression level during infection. This might indicate that the pathogenicity of *Colletotrichum* strains could be enhanced by actively transferred pathogenic factors located on mini-chromosomes. Although this gene (CASFJ_18279) was not necessarily typical of genes on mini-chromosomes, the association with pathogenicity of mini-chromosomes in *Colletotrichum* genomes was investigated at single-gene resolution level for the first time. Individual gene knockout and the presence of numerous pathogenic genes on mini-chromosomes of *C. asianum* FJ11-1 provided evidence that strain-specific mini-chromosomes were potential carriers of virulence factors. Further research is needed to investigate this relationship, as well as the origin and function of mini-chromosomes in other fungi.

## Data Availability

Illumina sequencing data for RNA-seq have been submitted to the National Center for Biotechnology Information (NCBI) Sequence Read Archive (SRA) under BioProject: PRJNA872313. The assembled genome sequences of 17 Colletotrichum strains have been submitted to NCBI genome database under BioProject: PRJNA872318. The source code used in this study are available on GitHub.

## SUPPLEMENTAL MATERIAL

Fig S1 mbio.00629-23-s0001.tif Syntenic plot. Global view of synteny alignment of mango anthracnose dominant strains. Only the top ten chromosomes are shown. GD10-1 served as a reference genome (in light orange). The red rectangles display the syntenic blocks, and the chromosome numbers are given on the blocks. +, Watson strand; −, Crick strand. Grey bars denote scaled PacBio reads coverage. Red dots display telomeric repeat. Green bars denote DNA repeats.

Fig S2 mbio.00629-23-s0002.tif (A) The Ultrametric tree of *Colletotrichum* genus. (B) The insertion age of intact LTRs in *Colletotrichum*.

Fig S3 mbio.00629-23-s0003.tif Gene syntenic plot of non-CGSC strains (CTR, CSP, CAC, CDE, CHI, CLE represent *C. truncatum* MTCC-3414, *C*. sp.JS-367, *C. acutatum* KC05, *C. destructivum* YC1, *C. higginsianum* IMI349043, *C. lentis* CT-30 respectively). Grey ribbons represent genes and red dots represent telomeric repeat.

Fig S4 mbio.00629-23-s0004.tif Pathogenicity and virulence tests with CASFJ_18279 mutants and complementation strain. (A) Lesion diameters of mango fruit. (B) Lesion diameters of mango leaves. (C) Virulence symptoms of mutants. (D) Virulence symptoms of complementation strain.

TABLE S1 mbio.00629-23-s0005.xlsx Summary of PacBio sequencing details for 17 isolates of different *Colletotrichum* spp.

TABLE S2 mbio.00629-23-s0006.xlsx Genome assembly details of *Colletotrichum* strains sequenced in this study.

TABLE S3 mbio.00629-23-s0007.xlsx Strand-specific RNA-seq reads mapping information of mango anthracnose dominant strains at different stages.

TABLE S4 mbio.00629-23-s0008.xlsx Features of mini-chromosomes.

TABLE S5 mbio.00629-23-s0009.xlsx Gene function of HGT genes from bacteria.

TABLE S6 mbio.00629-23-s0010.xlsx Summary of primers used in this study for FJ11-1 CASFJ_18279 gene knockout and complementation assay.

ASM does not own the copyrights to Supplemental Material that may be linked to, or accessed through, an article. The authors have granted ASM a non-exclusive, world-wide license to publish the Supplemental Material files. Please contact the corresponding author directly for reuse.
