# Supplementary figures and images for: The evolution of mini-chromosomes in the fungal genus Colletotrichum
Source: mBio. 2023 Jun 7;14(4):e00629-23. doi: 10.1128/mbio.00629-23 (PMC10470602; doi:10.1128/mbio.00629-23)

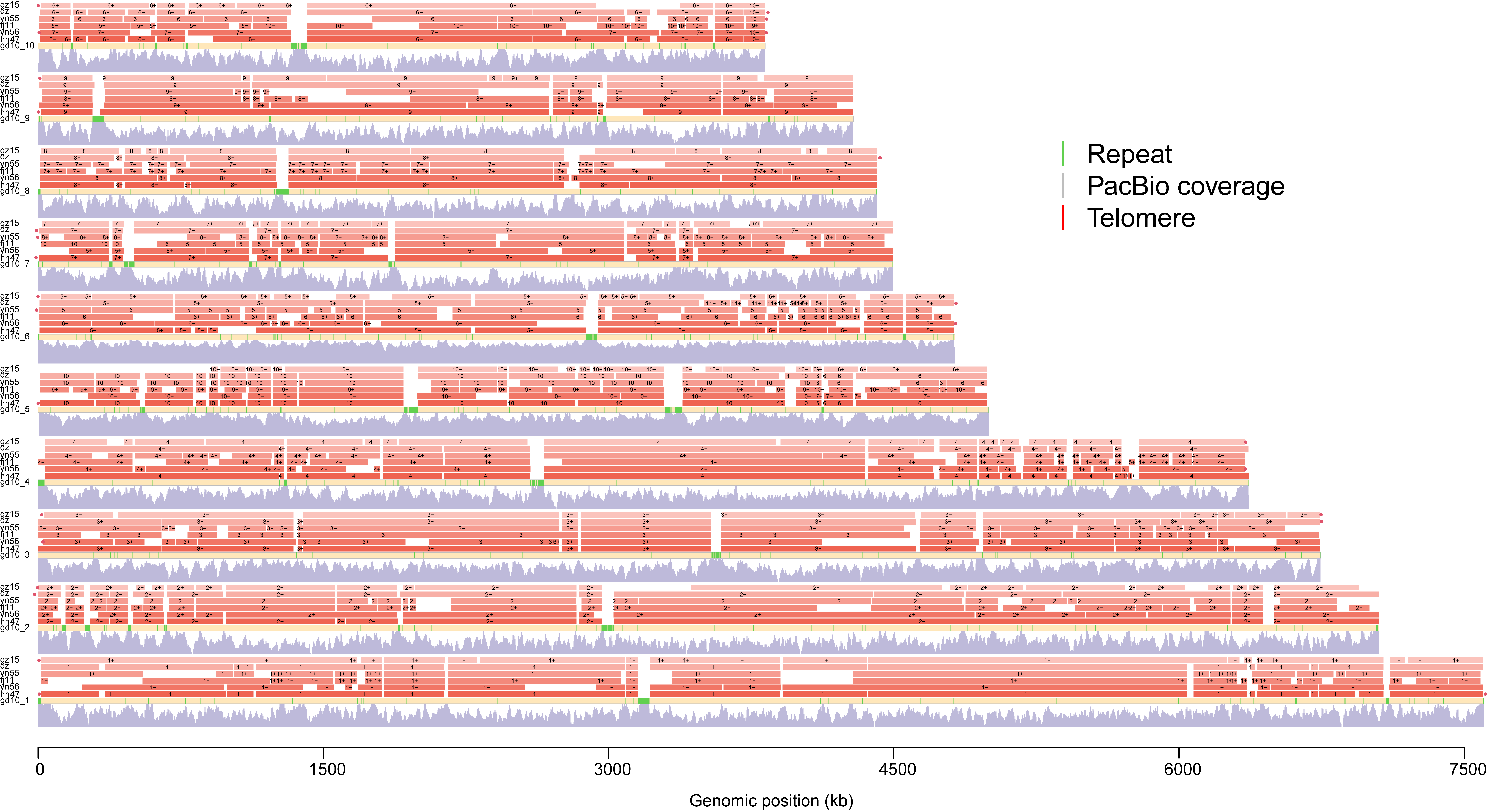

Supplement: Fig S1 — Syntenic plot. Global view of synteny alignment of mango anthracnose dominant strains. Only the top ten chromosomes are shown. GD10-1 served as a reference genome (in light orange). The red rectangles display the syntenic blocks, and the chromosome numbers are given on the blocks. +, Watson strand; −, Crick strand. Grey bars denote scaled PacBio reads coverage. Red dots display telomeric repeat. Green bars denote DNA repeats. [file mbio.00629-23-s0001.tif]

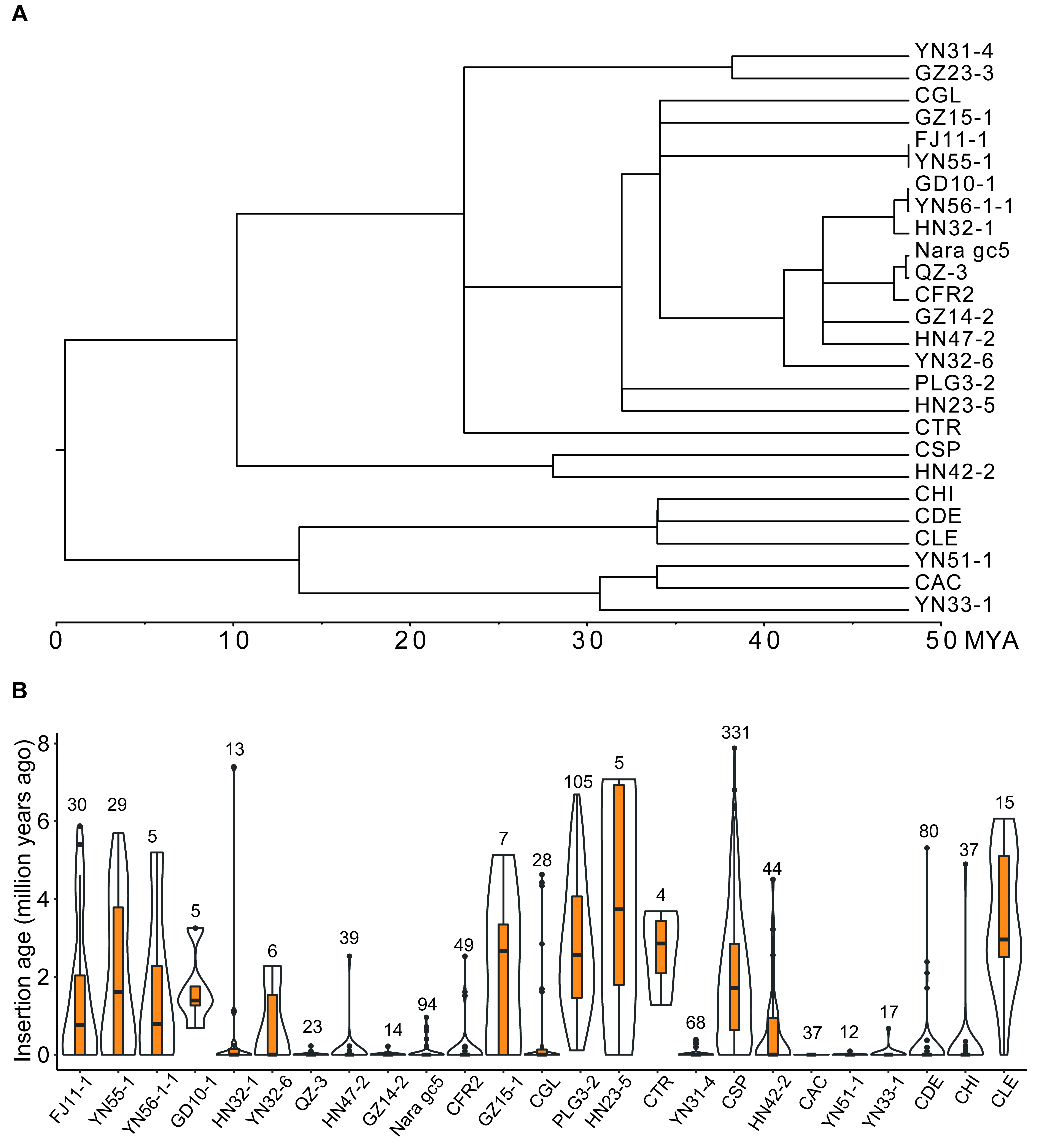

Supplement: Fig S2 — (A) The Ultrametric tree of Colletotrichum genus. (B) The insertion age of intact LTRs in Colletotrichum. [file mbio.00629-23-s0002.tif]

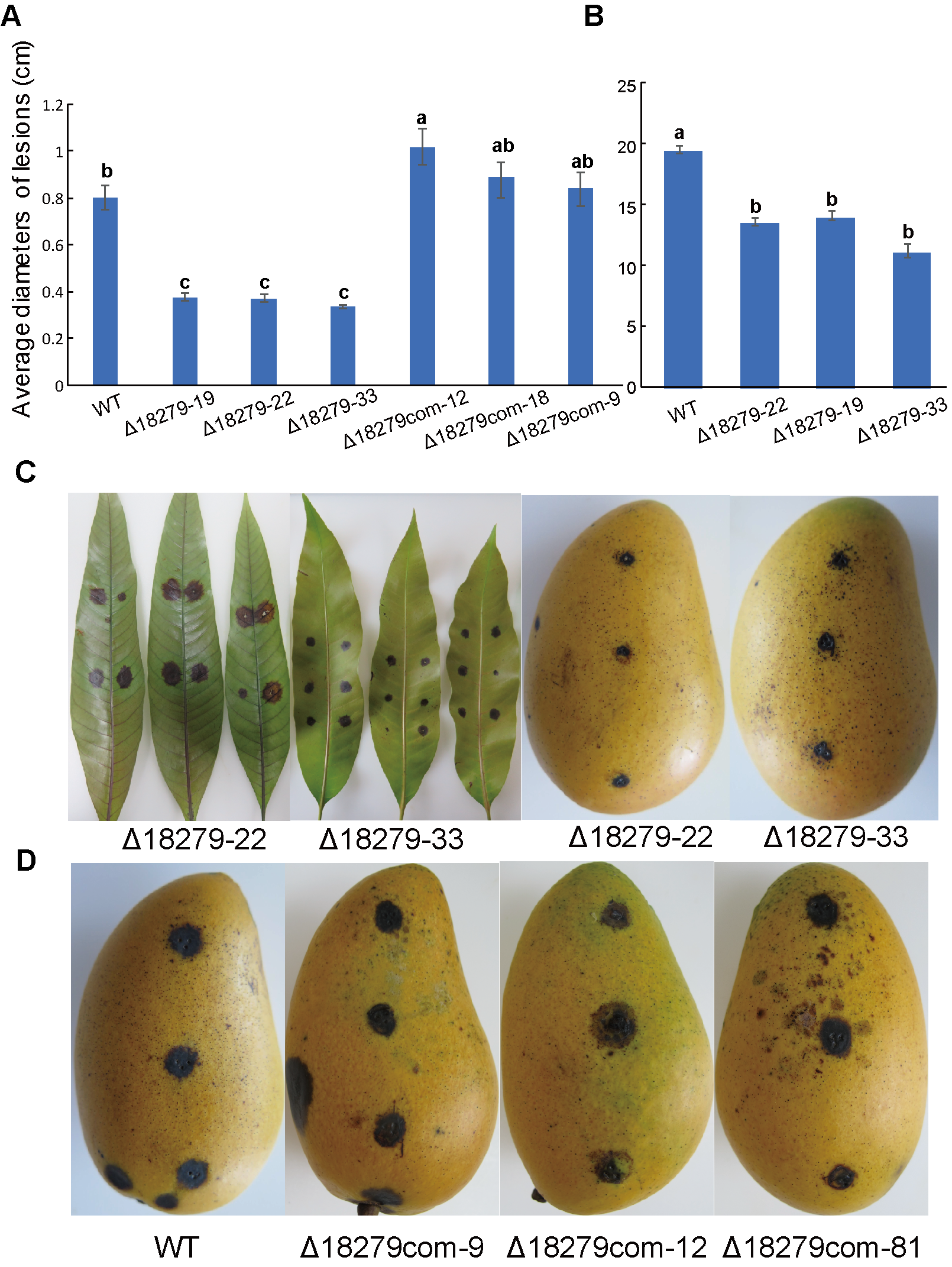

Supplement: Fig S4 — Pathogenicity and virulence tests with CASFJ_18279 mutants and complementation strain. (A) Lesion diameters of mango fruit. (B) Lesion diameters of mango leaves. (C) Virulence symptoms of mutants. (D) Virulence symptoms of complementation strain. [file mbio.00629-23-s0004.tif]
